# Heat-health vulnerability in temperate climates: lessons and response options from Ireland

**DOI:** 10.1186/s12992-020-00554-7

**Published:** 2020-03-30

**Authors:** Shona K. Paterson, Christie Nicole Godsmark

**Affiliations:** 1 College of Business, Arts and Social Sciences, Brunel University London, Uxbridge, UB8 3PH UK; 2 School of Public Health, University College Cork, Western Gateway Building, Western Road, Cork, T12 XF62 Ireland; 3 Environmental Research Institute, University College Cork, Cork, Ireland

**Keywords:** Climate change adaptation, Heat-health, Vulnerable, Temperate climate, Environmental health

## Abstract

**Background:**

In Ireland, rising temperatures remains the climate projection that national climate scientists associate with the highest degree of confidence. However, the health challenge of heat has been largely absent from Ireland’s public health sector. This is epitomised by the lack of a comprehensive public health-focused heat-health action plan or country-specific codes of practice for heat-health when working outdoors. Our objective is to highlight the anticipated heat-health challenges in Ireland, and other temperate regions, through analysing vulnerable groups and systems, reinforcing the need to respond.

**Methods:**

A scoping literature review was conducted to determine how heat affects health of the vulnerable in temperate climatic regions, with a focus on Ireland. Additionally, national Google Trends data was coarsely analysed to determine whether heat is a growing societal concern.

**Results and discussion:**

The heat-vulnerable include: older people; chronically ill; infants, pregnant women, children; outdoor workers; socio-economically disadvantaged; urban dwellers; food systems and the health sector. Google Trends data suggest an increase in heat-related health searches over time, demonstrating rising levels of concern to temperature increases, reinforcing a gap in national policy associated with communication of, and response to, the heat-health challenge. Specific, actionable recommendations for adaptation and mitigation strategies are proposed.

**Conclusion:**

Heat poses a public and occupational health challenge, receiving limited attention in Ireland. Lack of a co-ordinated effort, places vulnerable populations at risk. Our recommendations, with reference to vulnerable groups and acknowledging the multi-sectoral nature of heat-health and climate change, advocate for the adoption of a “health and climate change in all policies” approach and the development of a public health-focused heat-health action plan.

## Background

The *United in Science* report [1] states clearly that the planet is at a tipping point and climate change is putting pressure on the ability to support and supply food, water, health and wellbeing for existing and future generations. This report, coupled with the Intergovernmental Panel on Climate Change (IPCC) special report on the impacts of global warming of 1.5 °C above pre-industrial levels, highlight the growing threat of heat as a major driver in the loss of ecosystem services as well as a major driver of human vulnerability [2]. The increasing nature of the threat is easily demonstrable with vulnerability to extremes of heat having steadily risen as indicated by an additional 220 million more people exposed to heatwave events in 2018 compared to 1986–2005 [3].

**Fig. 1:**
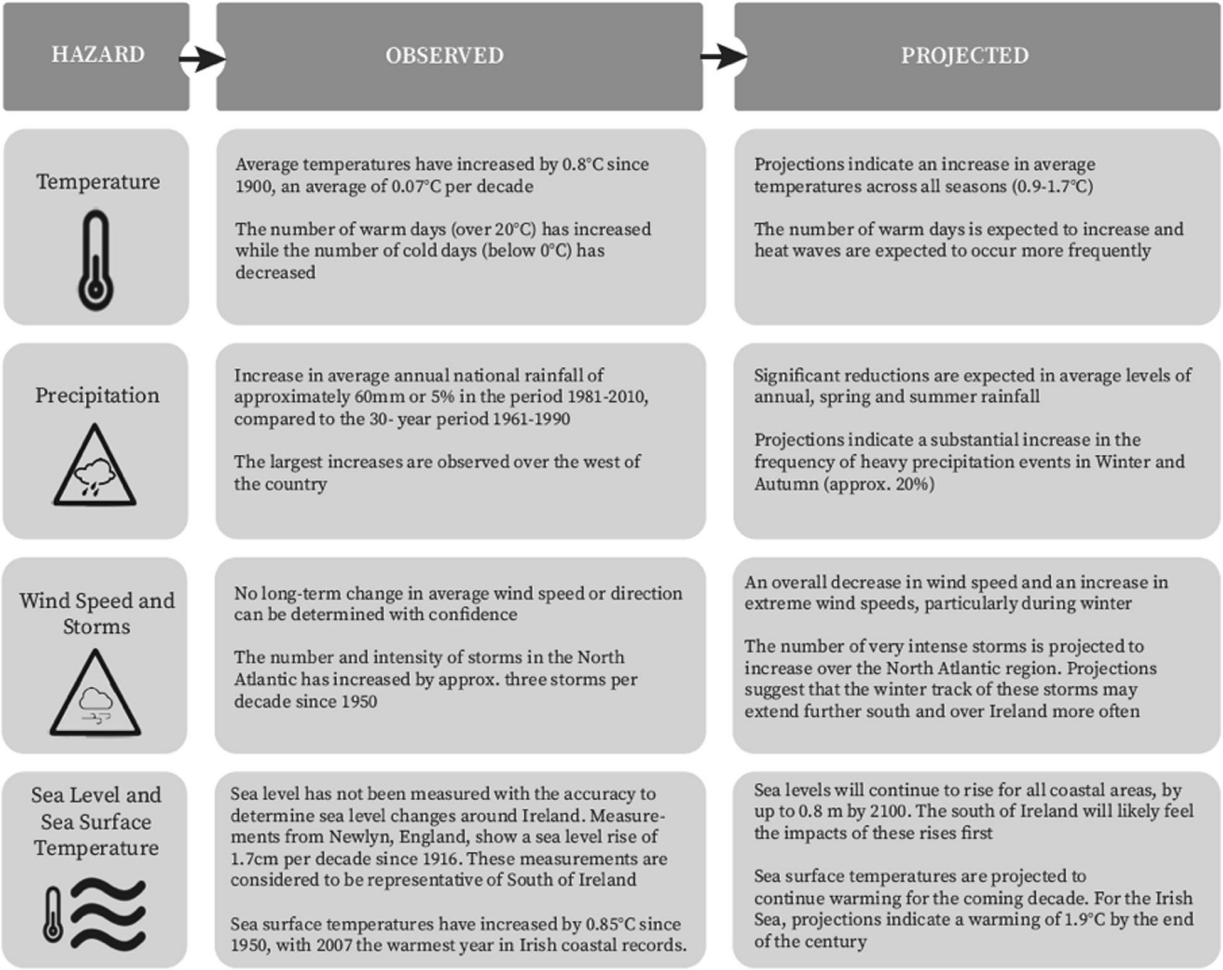
Observed and projected climate change in Ireland [4]

It is evident that changes in Ireland’s climate are already occurring and that the impacts visible today are expected to intensify in line with global projections over the coming decades. National climate projections include increased temperatures, disrupted precipitation patterns, changes in wind speed and direction, and increased storm frequency and intensity [4–7] (Fig. 1).

As with the IPCC’s Fifth Assessment Report (AR5) at a global level, the projections made with the highest degree of confidence by Irish climate scientists are that of rising temperatures and mean annual air temperature [8] with projected rises for Ireland ranging from 1.0 °C to 1.6 °C by 2050 with all seasons projected to be warmer by 0.9 °C to 1.7 °C by 2050 [4, 5, 9]. In addition, the occurrence and severity of heatwaves are projected to increase with a 10 to 40% increase in change in the percentage of summer (May–September) days classified as heat-wave days, and a 2 to 8% change in the maximum daily maximum temperature for days classified as heat-wave days [10]. Despite such high confidence statements in temperature projections, heat is yet to really be considered as a climate change-related health challenge in Ireland, a country currently classified as having a temperate climate (marine west coast climate, Cfb) under the Köppen climate classification system [11].

The Köppen climate classification system is a 5-type (A-E) classification that uses monthly temperature and precipitation to define boundaries of different climate zones around the world. This system has been employed in this paper to increase the viability of comparing impacts of, and responses to, heat across countries in a similar climate zone. With respect to this paper, the moist mid-latitude C and D climates which are broken into categories based on when the dry seasons occur in the zones as well as the coldness of the summer or the warmth of the winter, are most relevant for comparisons to Ireland.

### Impacts of heat

Globally, increased air temperature adversely affects health and increases morbidity and mortality [12–14]. This can occur through increased exposure to heat stress [12] but also, heat can exacerbate chronic conditions such as cardiorespiratory disease [15] and diabetes [16]. Additionally, indirect health outcomes can arise from increased temperatures such as an increased risk of violence [17], motor vehicle accidents [18], injuries [19], water-borne and vector-borne diseases [20, 21] (for a full review, see [8]). More recently, epidemiological advancements have been made linking increased temperatures with mental ill-health such as suicide [22], major depressive episodes [23] and other mental and psychosocial problems [24].

In alignment with repeated calls for greater preparation by temperate countries for the public health challenges of heat since the catastrophic 2003 heatwave in Europe [25], the 2019 Lancet Countdown report identified an increased vulnerability to heat especially within European populations when considering global burden of disease data [3]. During the last major heatwaves in Ireland (1983, 1984, 1995, 2003 and 2006) there were an excess of 294 deaths attributable to heatwaves [26]. However, assessing mortality attributable to heatwaves does not indicate the increased burden on the health sector from heat-related morbidity since these health outcomes are not isolated only to absolute extreme high temperatures but can also occur when temperature is relatively higher for a given place [27–29]. Currently, there appears to be no studies that comprehensively consider the heat-vulnerable amongst the Irish population. As the mean ambient temperature is projected to rise, with an increased number of warm days, it is also important to analyse heat-related mortality and morbidity during warm weather periods that may not be classified as a heatwave. The scientific evidence projecting an increase in global temperatures, coupled with the increasing vulnerability of Europeans, suggests that multi-sectoral actions are critically required to respond to this growing public health risk.

## Methods

The overarching aim of this manuscript is to review the growing evidence of heat-health vulnerability in temperate regions, with a particular focus on Ireland, to identify a range of potential interventions that can begin to tackle this growing threat. Three objectives will achieve this aim: i) to conduct of a scoping review of populations vulnerable to heat within the context of temperate climates focusing on Ireland, where evidence is available; ii) to briefly investigate the trend of the Irish populations’ heat-health concern over time through an analysis of Google Trends data; iii) to provide potential adaptation and mitigation options for an appropriate response to heat-health in temperate regions relating to the heat-health vulnerabilities identified in the review. This work, and particularly the recommendations proposed, is largely applicable to other countries with temperate climates with Ireland being used as a case study example.

### Scoping review

A scoping review of existing literature on heat-health in Ireland was conducted using PubMed, Web of Science and Google Scholar (first 50 results) databases in January 2020 using keywords: heat; health; Ireland. Inclusion criteria included: published in English; pertaining to Ireland (i.e. using Irish data or concerning the Irish population); with the primary aim of exploring the impact of climate-related heat on human health (e.g. excluding fuel poverty and household heating in cold weather, excluding food-processing or heat-treatment of food before consumption); studies involving humans (e.g. excluding studies on human cells and heat shock proteins). PhD theses and articles introducing projects were excluded. This search resulted in the total retrieval of 15 papers after reviewing for relevance and removing duplicates. Next, the most recent IPCC report, Chapter 11 Human Health: Impacts, Adaptation, and Co-Benefits, Sections 11.3 (Vulnerability to Disease and Injury Due to Climate Variability and Climate Change) and 11.4.1 (Direct Impacts of Climate and Weather on Health: Heat- and Cold-Related Impacts) [8] were reviewed for the identification of the heat-vulnerable. The following were identified as possessing vulnerability to heat and were used to structure the review: older people; chronically ill; infants, pregnant women and children; outdoor workers; socio-economic status and urban dwellers; food systems and the health sector. Where there was no literature available for the Irish context for a given vulnerability, a systematic search on PubMed was conducted using the keywords: heat; health; [vulnerability]; [temperate climate region classified using the Köppen system closest to Ireland e.g. United Kingdom, France]. Given the global dependencies and the complexity of the impact of heat on food systems and the health sector, a brief narrative review was conducted for those sections.

### Google trends analyses

A rapid analysis of Google search data from regional internet search frequencies (RISFs) was conducted to analyse the public’s Google search frequency of heat stroke over time. Google Trends data for Ireland was limited to Dublin by the software. Households in Dublin have the highest internet access (fixed broadband) in the country at 90%, with 84% of people accessing the internet every day in Dublin [30]. The term “heat stroke” was inputted into Google Trends, the geographic location was narrowed to Ireland, the timeframe was selected as the past 5 years, the “Health” category was selected, and results were limited to web search. RISFs displayed on Google Trends indicate the frequency of the specific search term (in this case heat stroke) as a proportion of all other searches. This is normalised between 0 and 100 providing the indication of frequency. A value of 100 is indicative that at the point the search for the term was the greatest over the time period measured for the specific location selected.

## Results and discussion

Our scoping review revealed a paucity of literature regarding heat-health in Ireland, identifying 15 papers that met the inclusion criteria which are discussed in the relevant sections below. As Irish literature was not found for every heat-vulnerability, additional literature from global studies are discussed to provide overall context with additional literature from other temperate climates to provide more specific context. Of the 15 relevant papers for inclusion, three focused on Ireland specifically and 12 included Ireland as part of multi-country studies. A review of this literature provides a mixed picture of potential impacts as well as major limitations in current research. For instance, many multi-country studies did not consider future population projections and vulnerability parameters and reported largely on mortality and not morbidity which likely masks the full burden of heat on health.

Several studies provided evidence to suggest that high temperatures do pose a public health risk globally. Guo et al. [31] suggested that mortality displacement (harvesting effect) can be viewed from an epidemiological analysis in Ireland, while Armstrong et al. [32] concluded that heat can result in mortality displacement but that for Ireland, the attributable fraction is low (however, it must be considered that with ambient temperature change, the prevalence of exposure is high). Multi-country studies that have taken into account population change scenarios have shown that in the face of no future heat adaptation, it is likely that countries in temperate zones will see an increase in heat-related excess mortality, even if this is not as large as projected for countries with hotter climates [33]. Additionally, Fronzek et al. [34] state that heat-related mortality is likely to increase for the region of the British Isles in response to a warming climate and population changes.

A second category of papers demonstrated no change in heat vulnerability over time. Baccini et al. [35], for example, found no deaths attributable to summer heat in Dublin alone over an 11-year period. However, there is evidence that there was limited variation in summer temperatures over this period [36], a reality that is expected to change under current climate projections. Not all manuscripts indicated that heat would result in poorer health outcomes for the Irish population [35, 37, 38]. Vicedo-Cabrera et al. [37] projected that excess deaths in Ireland may actually decrease due to the reduction in cold-related deaths when modelling projected changes in net excess mortality with a 1.5 °C vs 2 °C global mean temperature increase. However, changes in population or future vulnerability were not accounted for in the model as the authors acknowledge. Gasparrini et al. [39] also identified a similar trend of negative net effect of excess deaths for Northern Europe but mentioned that this trend would likely reverse toward the end of the century. Again, the limitation of not accounting for population changes was acknowledged.

### Heat-vulnerable populations

Whilst the entire population will be exposed to ambient heat stress, certain individuals are more vulnerable to heat and require a targeted adaptation response.

#### Older people

Globally, older people are regarded as being particularly vulnerable to heat [40] and the World Health Organization (WHO) project an additional 38,000 older people will die every year globally due to heat exposure [41]. In the global meta-analysis conducted by Bouchama et al. [42], death was more likely during a heatwave for those individuals unable to care for themselves (odds ratio [OR]: 2.97 [95% confidence interval [CI]: 1.8–4.8]), confined to a bed (OR: 6.44 [95% CI: 4.5–9.2]) or do not leave their home (OR: 3.35 [95% CI: 1.6–6.9]); behaviours characteristic of some older people. Mortality and morbidity statistics related to increased temperatures for older people are unknown for Ireland despite 13.4% of the Irish population being over the age of 65, an age group that has been increasing since the 1980s [43]. Roche et al. [44] in their analysis of computer tomography kidney, ureters and bladder scans in an Irish hospital group, did find that older adults (> 65 years) were more susceptible to ureteral colic during warm weather compared to those below 65 years old, potentially due to an increased susceptibility to dehydration. Older adults (> 75 years) were identified as a particularly vulnerable group in Pascal et al.’s [26] study of heatwaves and mortality in Ireland. Analysis from General Practitioner consults and Emergency Department visits in England (Köppen system classification: temperate, Cfb) indicated that during the 2013 heatwave, older people (aged ≥75 years), along with school children, were particularly at risk of heat illness [45]. Notably, ill-health continued after the heatwave ended in the ≥75 years age group [45]. Despite WHO/Europe recommendation for the implementation of heat-health action plans [46], only limited heat response planning has been conducted by relevant bodies in Ireland such as weather warnings from Met Eireann, the Irish meteorological service, and press releases and website information from the Health Service Executive (HSE), Ireland’s community and hospital public health service.

#### Chronic conditions

A compounding factor is that a large proportion of older people take medication for chronic conditions. It is estimated that 33% of over 65 s in Ireland report polypharmacy (regularly taking ≥ five medications), with medications for cardiovascular disease and hypertension the most common [47]. Although a topic currently under-researched, medication use can increase vulnerability to suffering heat-related illness. Anticholinergics, antihistamines, phenothiazines, tricyclic antidepressants and antispasmodics can impair sweating; tricyclic antidepressants and ergogenic stimulants can alter heat production; lithium, diuretics, laxatives and levothyroxine disrupt water balance within the body; whilst calcium channel blockers, barbiturates and beta blockers are known to alter skin blood flow and / or blood pressure [48]. Even non-steroidal anti-inflammatory drugs (NSAIDs), such as aspirin and ibuprofen, can alter core body temperature and efficient thermoregulation [49]. Indeed, Page et al. [50] in their study on mental illness and temperature-related deaths in England (Köppen system classification: temperate, Cfb) found that taking hypnotic/anxiolytic and antipsychotic medications increased the relative risk of mortality during hot weather by approximately 7% per 1 °C temperature increase, although the association did not seem to hold true for taking antidepressants. A comprehensive review, focusing not only on older people, is required on the implications of medication usage in temperate regions in the context of a warming climate and the current burden of disease.

It is not only older people taking medications that are vulnerable but with the high burden of chronic non-communicable diseases (NCDs) in Ireland, and many other temperate regions, many more could be at risk. According to the WHO, globally, 80% of all premature NCD-related deaths occur from cardiovascular disease, cancers, respiratory disease and diabetes [51]. A global meta-analysis conducted by Bouchama et al. [42] calculated that suffering from cardiovascular disease or pulmonary disease increased the odds of death during a heatwave by 2.48 (95% CI: 1.3–4.8) and 1.61 (95% CI: 1.2–2.1) respectively. In Ireland, Pascal et al. [26] found excess deaths during heatwaves attributable to cardiovascular and respiratory causes. Furthermore, analyses from the UK (Köppen system classification: temperate, Cfb) also found an increased sensitivity for heat-related deaths by cause of cardiovascular or respiratory disease [52]. In a study conducted by Rey et al. [53] the greatest percentage of excess mortality during six heatwaves between 1971 and 2003 in France (Köppen system classification: majority temperate, Cfb) were observed for cardiovascular, respiratory diseases as well as neoplasms, heat-related causes, injuries and poisoning. All neoplasms (i.e. cancers) were included in the analyses (ICD-10, C00-D48). Type-2 diabetic and obese individuals are also at risk of ill-health during high ambient temperatures. In a temperate region (Köppen system classification: temperate, Cfb) English type-2 diabetic patients were found to be at an increased odds of presenting to their medical practitioner on days of high ambient temperature and these odds were elevated for diabetic patients with cardiovascular comorbidities [54].

Regarding other NCDs, in our Irish search of the literature, Roche et al. [44] found an association during the summer months in Ireland (Dublin), for an increased frequency of presentations to Emergency Departments for acute renal stones. Furthermore, a study conducted by Flaherty et al. [55] suggested that specific travel advice should be provided to obese people that may exhibit poor heat tolerance travelling to warm climates. NCDs in Ireland are the greatest cause of death accounting for 575.36 deaths per 100,000, with nine of the top 10 DALYs attributable to NCDs [56]. With such a large burden of disease attributable to NCDs, further determination of the impact of heat on the health of these populations should be urgently conducted in Ireland so as to respond to what could be an emerging, compounding health crisis.

The mentally ill are particularly heat susceptible as the global literature identifies due to medication usage, a limited ability for self-care, poor cognitive awareness of environmental hazards and inadequate adaptive behaviour change such as hydration and clothing choice [28, 42, 48, 57, 58]. This relationship has been identified in a temperate city, Adelaide (Köppen system classification: temperate, Csa), as shown by a 7.3% increase in hospital admissions for psychological disorders during heatwaves [28]. It is estimated that those with a psychiatric illness are at a triple risk of death during a heatwave (OR: 3.61 [95% CI: 1.3–9.8]) and taking psychotropic medication increases the likelihood of death almost two-fold (OR: 1.90 [95% CI: 1.3–2.8]) [42]. Mental ill-health in Ireland carries a prevalence of 18.5% including anxiety and depressive disorders, bipolar disorders and schizophrenia as well as alcohol and drug use disorders [59]. The prevalence of mental ill-health in the Irish population is higher than the EU average of 17.3% and only slightly lower than Finland, which exhibits the highest prevalence in the EU at 18.8% [59]. Given this prevalence of mental ill-health, along with subsequent medication usage to manage disorders, the effects of heat on populations with poor mental health in temperate regions needs further investigation.

#### Infants, pregnant women and children

Heat stress can affect human health and wellbeing throughout the lifecycle including the unborn child, infants, children and pregnant women [60, 61]. A global systematic review by Zhang et al. [61] found that both high and low temperatures were associated with adverse birth outcomes of pre-term birth, low birth weight and sometimes, stillbirth. The association appeared stronger for high temperatures however the authors called for more research to be undertaken as well as further research in “diversified climate zones” as the review had no geographical boundaries. Epidemiological studies of temperature effects on birth outcomes have been conducted in temperate climates (Köppen system classification: temperate, Cfb) such as London [62] and Aberdeen [63]. Results are mixed, with the London study [62] reporting a non-significant effect for maximum temperature and preterm births, and the Aberdeen study [63] reporting reduced birthweight if the ambient temperature was high during the first trimester of pregnancy. Therefore, we concur that more studies are needed in different climate zones, particularly in temperate regions, to determine the effects of high ambient temperature for a given location on birth outcomes.

As indicated in a study from Melbourne, Australia (Köppen system classification: temperate, Cfb), during warm months, children might desire to spend more time outdoors [64], thus potentially exposing them to environmental heat stress, UV radiation and air pollutants through this behaviour change [65, 66]. Physiologically, there is debate whether children are vulnerable to heat stress through underdeveloped and reduced sweating responses [65, 67–69], reduced locomotion economy resulting in greater heat production per unit of body weight [68], smaller total blood volume [70] and increased requirement for shunting cardiac output to the skin compared to adults [68]. Although children’s reliance on dry heat dissipation may mitigate their vulnerability [68]. Behaviourally, it is generally accepted that children are vulnerable to heat stress given their decreased awareness of fluid intake requirements [65], a possible extended duration to achieve acclimatization [71], spending time outdoors in play as well as often playing on, or close to the floor where thermal radiation levels can be high depending on solar intensity and surface material.

Although research is sparse and outdated, it has also been suggested that poor regulation of the indoor environment, including high temperatures, can result in lowered test performance in children [72]. Certainly, high temperatures indoors have been shown to reduce adult job speed, concentration and clarity of thought [73, 74]. The impact of air-conditioning regulating the indoor environment to a tolerable thermal comfort level could mitigate temperature-induced performance decrements [75, 76] however, research on this is limited, outdated and requires further investigation, particularly over the loss / gain of an increased energy requirement for air-conditioning and possible emissions.

#### Outdoor workers

Outdoor workers, those who are physically active and those who are recreationally active outdoors are also considered heat-vulnerable. Outdoor workers are at particular risk to six out of the seven climate-related occupational hazards identified by Schulte et al.’s [77] framework. That being: increased ambient temperature; air pollution; ultraviolet radiation; extreme weather; vector-borne disease and other biological hazards; industrial transitions and emerging industries (e.g. green jobs). Of note is outdoor workers required to wear personal protective equipment (PPE) who are at an elevated risk of suffering heat-related illness. Under specific conditions, heat strain can even occur in temperatures as low as − 20 °C (if the workload is moderate to high and the worker is wearing semi-permeable or impermeable PPE [78–80]). Although gender and heat-vulnerability has not been well characterised in the literature, it may be that occupational status may be a relevant factor in many studies suggesting a heat and gender-vulnerability association, usually placing males at greater risk [8]. Indeed, in a study of acute nephrolithiasis during summer in Ireland, men were found to be at greater risk of developing stones in warm weather compared to women [44]. However, in a global study focused on women firefighters, there is evidence to suggest that they suffer from heat-related illness at work with the call for further specific heat-related support for this heat-vulnerable population [81].

A study on non-military working populations in the state of Washington (USA) (Köppen system classification: temperate Csb, Csc, Cfb) found that heat-related compensation claims for workers were associated with high ambient temperatures [82]. Common occupations associated with the claims included: construction workers, fire fighters, labourers and material movers. Other studies from the region have indicated adverse heat-related health outcomes for agricultural workers [83]. Research on climate change-induced warming on occupational health in outdoor workers in Ireland does not yet exist to our knowledge, despite over 10% of Ireland’s workforce currently employed in common outdoor sectors of agriculture, forestry, fishing and construction [84]. Although ISO standards (International Organization for Standardization) exist, Irish-specific policies do not appear to adequately protect the outdoor worker from heat-related adverse health outcomes. For example, to our knowledge, there are no Irish-specific codes of practice for working outdoors in extreme heat. A useful example to adapt to the Irish situation would be the National Institute for Occupational Health and Safety’s (NIOSH) Criteria for a Recommended Standard: Occupational Exposure to Heat and Hot Environments. Another example, although not as comprehensive, is the United Kingdom’s Health and Safety Executive’s guide: Heat Stress in the Workplace - A Brief Guide; as well as various resources from the UK Health and Safety Executive’s website. The development of such policies by the Irish Health and Safety Authority is essential for a proactive approach, rather than reactive response to the occupational health challenge of heat in the workplace. Financially, evidence has shown that the costs of implementing systems proactively to protect against the risks of climate change on health will save costs in the long-term as compared to reacting to incidents [14, 85]. Global statistics are available: an estimated 153 billion hours of labour were lost in 2017 due to a warming planet, 80% of which was from the agricultural sector [86] projected working hours lost by 2030 due to heat are 2% per year [87]. Thus, the opportunity exists for Ireland to adopt evidence-based proactive action on protecting outdoor workers from heat.

Whilst Pascal et al. [26] identified and analysed mortality attributable to heatwaves for vulnerable groups in Ireland, such as older people and those living in rural or urban areas, a further analysis on vulnerable groups was not undertaken although the authors mention the importance of focusing on outdoor workers including farmers. We would echo this call for research prioritization of farmers and would also add other vulnerable outdoor workers of specific relevance to Ireland such as those required to wear PPE and construction workers. Particularly as the construction sector is experiencing steady growth in Ireland following recovery from the recession. It is essential that health and safety policy specific to Ireland, and other temperate regions, include basic guidelines for employers to adhere to workplace heat limits and surveillance, hydration strategies, mandatory rest breaks during hot weather and special considerations for those wearing PPE.

We also advocate that a needs assessment be undertaken to determine training and capacity needs and research priorities for outdoor workers leading to improved health and safety. Adam-Poupart et al. [88] provide a comprehensive overview of the main impacts of a changing climate on occupational health and safety in Quebec, Canada (Köppen system classification: continental Dfa, Dfb, Dsc and polar ET). Heat was identified as a key hazard with the following potential consequences on occupational health and safety: heat-related illnesses (including cardiovascular and renal issues, dehydration); increased absorption of chemicals; reduced cognitive performance; dermatological issues; increased accidents related to lowered vigilance and manual dexterity; perturbed emotions and mortality [88]. Priority focus areas for research included the interactions of chemical toxicity and heat as well as the physiological strain of wearing PPE during high temperatures [88]. It was also recommended that heat-health-focused training tools be developed and disseminated [88]. Investigation of heat-related risks, research priorities and recommendations for improved occupational health and safety, are also needed for Ireland.

#### Socio-economic status and urban dwellers

Further factors compounding vulnerability to heat stress, even in temperate climates includes those of a poor socio-economic status [89] and those living in urban heat islands (UHI) [90]. In an analysis of the European heatwave in 2003, Rey et al. [89] identified that in Paris (Köppen system classification: temperate, Cfb), those who were deprived were more vulnerable to death during the heatwave. Vulnerability of the poor to heat may be through reduced adaptive capacity such as the inability to afford air conditioning, poor general health, lifestyle risk factors and living in densely populated cities. Although race and ethnicity and heat-vulnerability has not been well characterised in the literature, it may be that community-level characteristics may be a relevant factor in many studies suggesting a race or ethnicity-vulnerability association [8]. Although the most recent Survey on Income and Living Conditions (2017) in Ireland revealed that overall, poverty and deprivation is decreasing as the mean annual household disposable income rose by 4.7% from 2016; the “at risk of poverty rate” was at 15.7% of the total population [91]. This indicates the percentage of the population “whose equivalised income was less than 60% of the national median equivalised income”.

Heaviside et al. [90] assert that the projected impact of heat on populations is likely underestimated given that temperature monitoring stations are usually on the outskirts of heavily built up areas. With high spatial resolution modelling over two different scenarios (one taking into account the UHI), Heaviside et al. [90] estimated that the UHI contributed to 50% of the mortality during the August 2003 heatwave in the West Midlands of the UK (Köppen system classification: temperate, Cfb). Sheridan and Allen (2018) argue that even within cities, vulnerabilities differ spatially [92]. Internal migration statistics for Ireland indicate that with a 3.8% population increase between 2011 and 2016, the rural / urban divide grew during those years, with more people living in urban areas (62.7%) compared to rural areas (37.3%) [91]. Pascal et al. [26] caution that rural communities should also benefit from appropriate heat-health prevention plans, particularly in countries such as Ireland which has a significant rural population and where access to healthcare may be more remote. The long-term trend for Ireland is one of increased urbanisation, which from a heat-health perspective, translates to a steady increase in the proportion of the population at risk of heat-related morbidity and mortality.

### Heat-vulnerable sectors and systems

In addition to vulnerable populations, heat will impact sectors and systems in a variety of ways. This paper focusses on the two major systems that were identified during the course of the vulnerability review: the food (both marine and seafood, and agricultural) and health sector. The interdependency and multi-sectoral nature of these systems is recognised by several planning documents in Ireland including the National Adaptation Framework, the National Development Plan and Project Ireland 2040. For example, within the National Development Plan it is stated that “decisively responding to the challenges posed by climate change requires a whole-of-society and whole-of-government approach”. This combination emphasises the need for social outcomes and values to be evaluated alongside economic targets.

A brief discussion is presented below to illustrate not only the challenges and impacts of climate change, especially heat, on the food and health systems, but also the opportunities to develop specific, feasible and actionable recommendations for possible adaptation and mitigation strategies to ensure resilience within these systems. The following presents a brief narrative review on the impact of heat on food systems and the health sector.

#### Food systems

The direct and indirect impacts of climate change on the natural environment are expected to be significant in the long term (2050 and beyond), potentially further exacerbating existing pressures on ecosystems and contributing to the further decline of some species as well as impacting water, food production and land and soil erosion [93]. In particular, the natural environment is vulnerable to cascading effects that can potentially result in non-linear ecosystem responses, resulting in loss of ecological function and with major implications for human health and wellbeing. As function is reduced or lost, the buffering the natural environment provides against hazards such as top soil erosion or pests, is sharply reduced.

Ireland’s National Adaptation Framework (2018) states that both the agricultural and the marine and fisheries sectors will be deeply affected by climate change [7]. Changes in air and soil temperatures and changes in rainfall patterns and extreme events are predicted to cause water stress for crops and heat stress for animals. In addition, plant diseases which are currently rare may occur more frequently and the mobility of machinery on fields may be affected due to increased levels of winter rainfall. Increased sea surface temperatures will affect the biogeographical ranges of species distribution including major commercial fish stocks. Warmer waters support lower levels of dissolved oxygen and provide favourable conditions for the growth of algal blooms resulting in increased health risks [7]. In addition to direct impact such as heat stress on food systems, there is an increasingly growing global literature on the impacts of climate change on food insecurity (e.g. [94]), food-related illnesses (e.g. [8]) and the loss of nutritional value of food (e.g. [95]). This combination of impacts will have wide ranging implications at the global scale as well as the national one.

Linked closely with the vulnerability of outdoor workers, the Irish food system, with a large focus on beef and dairy production, is particularly vulnerable to rising temperatures. Just as humans have a physiological thermal tolerance limits, so too do crops and livestock. In the temperate climate of the UK, Part et al. [96] found that broiler chickens were vulnerable to heat stress-induced mortality during warm and humid weather. Palacio et al. [97] found financial benefits (decreased water consumption) when pasture dairy cows were provided with shaded areas during the summer in a temperate climate (Ontario, Canada). The call for providing livestock shelter in warm weather of temperate climates was also advocated by Moons et al. [98] for improving welfare and productivity. Initial reporting into the impacts of climate change on dairy production in Ireland have identified extreme weather events, fodder production challenges and the emergency of new diseases and pests as major threats but did not identify heat specifically as a challenge to the industry [99]. This suggests that the industry is either not yet aware of the issue or that sufficient evidence has not yet been provided for sector decision-makers.

Global estimates of the impact of heat stress on crop yields (wheat, maize, rice and soybean) found that a particularly sensitive region globally were continental regions between 40° and 60° North [100]. Although Ireland’s location is at 52° N, it is not continental and may not be at the highest risk. However, warming temperatures, often in conjunction with increased wetness, provide more suitable habitats for growth of biological contaminants [101] and it is projected that globally increasing temperatures might introduce new pests and pathogens [102]. All of these changes would result in a greater need for pest and contaminant control, which is often in the form of chemicals (pesticides, herbicides, fungicides etc.). This will increase the chemical load on ecological systems and increases the risk of hazardous chemical exposure and poisonings in humans. Indeed, Patterson et al. [103] state that another concern is how climate change may affect the potency or effectiveness of pesticides used for pest control and project a possible further increase in chemical use because of this. It is not only the direct health impacts of potentially greater exposure to chemicals that is a concern for farmer-health, but also the mental distress possibly experienced in light of the financial stress arising from these, and other climate-related complications.

#### Health sector

The health and social care sector is a large employer in Ireland. While it forms a significant component of the economy, it is also recognised as a source of carbon emissions. Therefore, the sector must play a dual role: to lead by example in acting to mitigate climate change as well as reduce the overall vulnerability of the population to the impacts of climate change. However, the sector itself is also vulnerable to rising temperatures from the perspective of healthcare quality and essential public health infrastructure and services such as power [8]. Most notably, this would be through an increased capacity need especially during extreme events. The projected increase in days of hot weather or heatwaves will likely increase the levels of morbidity and mortality exhibited in vulnerable populations both during and also following an event (lag structures) [15, 104]. This anticipated increase in service demand, dictates the need for a thorough analysis of hospital admissions during, and considering an admission lag after, the hot event, is necessary for detailed planning of particular care units or specializations that might require extra capacity such as geriatric, psychiatric and neonatal. Preparedness in supplies, capacity and other resources will involve further research of a detailed analysis of the anticipated health outcomes of rising temperatures to include both direct (such as dehydration) and indirect (such as food-borne diseases) health outcomes.

### Google trends analyses

The use of data from RISFs, through systems such as Google Trends, has been gaining momentum in healthcare research. Google Trends has been used for inferring causation, surveillance and descriptive purposes in health-related studies [105]. In the UK, it is estimated that approximately 81.8% of all internet searches use the Google interface, with the market share of desktop searches for Google being as high as 96.2% in Brazil and 95.9% in India [106], making it a relevant database to analyse. There are of course limitations with using such methodological techniques, for example excluding those who do not have a computer or access to the internet, low representativeness in key vulnerable groups such as older people, young and poor, limited geographical range of data collected and poor documentation of methods resulting in low reproducibility [105, 107]. Green et al. [107] analysed data from Google search patterns compared with syndromic surveillance systems in England to determine if such data could be useful in countries that lack sophisticated health surveillance systems to determine a potentially symptomatic population who do not access healthcare. Data from Google searches tracked closely with NHS 111 calls to which the authors concluded that Google search data could be utilized as a real-time surveillance system when there is no availability of more traditional health surveillance systems.

**Fig. 2:**
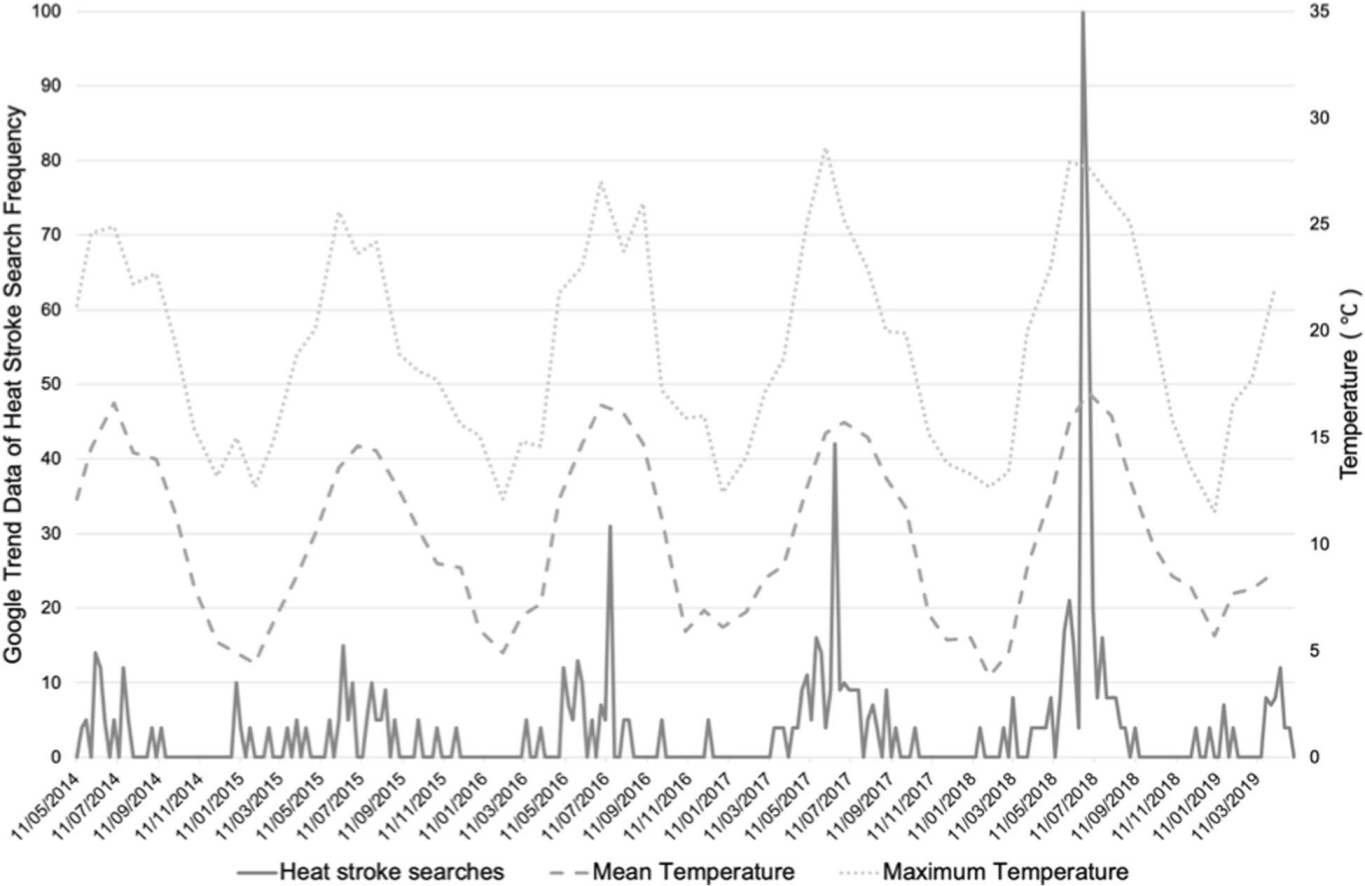
Mean and maximum temperature overlaid on Google Trends data for “heat stroke”. Temperature data (mean and maximum) from Phoenix Park Met Eireann weather station in Dublin overlaid on Google Trends data for the search term “heat stroke” in Dublin over the past 5 years. Note that an improvement to Google Trends data collection system was applied from 01/01/2016

The theme of this manuscript is highlighting the challenge that heat poses to public and occupational health in Ireland and also as a lesson to other temperate regions. With the argument that heat might not be given a large enough concern for the health sector given the limited policy, data and research on the topic despite the large proportion of vulnerable groups. To begin to uncover the argument that there is a lack of risk communication and public planning and awareness in Ireland on heat-health, it is useful to conduct a rapid analysis of Google search data (RISFs) regarding the health risks posed by heat. Google Trends data reveals that there has been an increase in searches overtime with a frequency peak reached toward the end of Summer 2018 (Fig. 2). A similar pattern is observed for the search terms of “heat exhaustion” and “heat rash”. Overlaying mean and maximum temperature for the same time period at a Dublin location (Phoenix Park weather station), tracks closely with the internet searches (Fig. 2).

This rapid analysis is inadequate to make any inference on health surveillance but does indicate clearly that public concern on heat-health is increasing. We argue that the health sector response to the challenges of heat on public and occupational health should be increasing too.

### How to respond?

Although evidence is incomplete for Ireland, we have shown the evidence-base of a number of public and occupational health challenges of heat for other temperate climates and thus, policy makers still require practical options for adaptation to heat for proactive action, rather than a reactive response. Table 1 was produced as a guidance for adaptation and mitigation planning options and strategies for a response to the public and occupational health challenge of heat in a temperate environment. No systematic methodological rigour was applied to the development of the Table, it is intended just as a guide of possible actions. The strategies in Table 1 draw from global activities and are not exhaustive although we make a number of specific recommendations within the domains of policy, planning, engineering, community, communication, capacity building and research priorities. It is essential that each potential recommendation be critically evaluated and appraised for any unintentional co-harms or maladaptation that may occur before being implemented.

**Table 1:** Potential adaptation and mitigation options for a response to heat in temperate regions

Vulnerable	Strategy	Potential Intervention or Response Option	Example of Responsible Entity
Older people, chronically ill and mentally ill	Communication	Develop and disseminate risk communication materials for older people, chronically ill and mentally ill (or carers) on coping mechanisms during heat events (e.g. increase fluid intake, improve building ventilation).	Health promotion unit of the health sector
		Communicate to patients the risks of heat to triggering or exacerbating chronic conditions such as cardiorespiratory diseases.	Doctors and General Practitioners (GPs)
		Communicate to patients the risks of heat interacting with medications with suggested associated behaviour changes.	Pharmacists, GPs, doctors, health promotion unit of the health sector
	Community	Develop and disseminate communication to the public on care for older people, chronically ill and mentally ill during heat events (e.g. check-in on neighbours and suggest / assist with coping options).	Health promotion unit of the health sector, community
	Capacity building	Provide targeted capacity building and refresher heat-health first aid courses for carers.	Health promotion unit of the health sector
	Planning & policy	Investigate financial assistance grants for home or common room (older people residential facilities) climate controls^a^ for the vulnerable (after a critical evaluation to avoid maladaptation).	Local government, government department responsible for climate action
	Research & Capacity building	Further research is required on these topics which should be followed with adequate capacity building to disseminate and translate research findings for evidence-based implementation: • Heat-related mortality and morbidity statistics for the temperate region, particularly with reference to the current burden of disease and those suffering chronic conditions (e.g. NCDs, mental ill-health) • Quantification of the extent of personal heat exposure in the homes of the vulnerable (e.g. older people) • The interaction of drugs, heat stress and thermoregulation • Quantification of older people, chronically ill and mentally ill access to green and blue spaces	Higher education institutions, research bodies, statistics body, government department responsible for health, funding bodies
Infants, pregnant women and children	Communication	Develop and disseminate risk communication materials for pregnant women, parents, teachers and carers regarding the risks of heat stress to children and infants (including the unborn child) where appropriate.	Health promotion unit of the health sector, GPs, doctors
	Engineering	Various engineering and planning controls could be developed as appropriate (after a critical evaluation to avoid maladaptation): • Investigate climate control^a^ in schools and improvements to ventilation (if needed).	Local government, government department responsible for climate action, government department responsible for education
	Capacity building	Provide targeted capacity building and refresher first aid courses for teachers and carers around heat-health in schools.	Health promotion unit of the health sector, government department responsible for education
	Planning	Provide the scope and discussion regarding children wearing hats to and from school during hot days.	Health promotion unit of the health sector, local government, government department responsible for education
		Re-schedule sports days during heatwaves and hot days.	Local government, government department responsible for education
	Research	Further research is required on these topics which should be followed with adequate capacity building to disseminate and translate research findings for evidence-based implementation: • Exposure of pregnant women to heat stress and the implications on mother and unborn child health in a temperate climate • Health impacts of the exposure to heat stress in children living in temperate climates • Benefits or risks of climate control^a^ in schools • Needs assessment of teachers and carers for heat-health in children	Higher education institutions, research bodies, statistics body, government department responsible for health, funding bodies
Outdoor workers	Capacity building	Develop and implement targeted heat-health capacity building, training and first aid (including self-monitoring and a buddy system) for outdoor workers and employers.	Government department responsible for health and safety, employers
	Research	Further research is required on these topics which should be followed with adequate capacity building to disseminate and translate research findings for evidence-based implementation: • The interaction of chemical toxicity (e.g. pesticides, herbicides) and temperature impacting worker health • Personal heat exposure of outdoor workers and those expected to wear personal protective equipment in temperate climates • Needs assessment of training and educational materials for heat-health amongst outdoor workers • A vulnerability assessment for outdoor workers working in hot conditions across sectors • Workplace surveillance of heat-related occupational injuries and mortalities • Safe exposure times and patterns specific to industries in temperate climates • Heat exposure and occupational health in temperate climates including mental health concerns of farmers and farm-workers	Higher education institutions, research bodies, statistics body, government department responsible for health, funding bodies
	Policy	The following should be critically evaluated for potential inclusion into health and safety policy: • Prepare a code of practice with guidelines and recommended standards for heat stress when working outdoors in Ireland, including a special focus on wearing PPE • Provide guidance on how to include heat-health into a risk assessment • Develop a heat alert programme activated when heatwaves are forecasted	Government department responsible for health and safety, employers
	Engineering & planning	Various engineering and planning controls could be developed as appropriate (after a critical evaluation to avoid maladaptation): • Use of fans (if appropriate) and heat shielding barriers • Re-organising work schedules and metabolic demands during hot times of the day • Introducing heat acclimatization plans if appropriate • Providing adequate rest breaks, shade and cool water on hot days • Organise and implement a medical monitoring programme (record keeping, surveillance, screening for heat intolerances) • Review the appropriateness of certain PPE and evaluate possible improvements • Provide engineered personal cooling garments, vests and devices if appropriate • Review and implement ISO standards as appropriate (e.g. ISO 7243, 9886, 15265, etc.)	Government department responsible for health and safety, employers, occupational health practitioners
Poor and urban dwellers	Communication	Provide risk communication to urban dwellers on the urban heat island and appropriate adaptive responses.	Health promotion unit of the health sector, local government
		Disseminate town / city maps noting all public drinking fountain locations.	Health promotion unit of the health sector, local government
	Community	Encourage a sense of community to provide assistance to the poor during heat events such as providing drinking water and protection from the sun and heat.	Health promotion unit of the health sector, community
	Planning & policy	Various engineering and planning controls could be developed as appropriate (after a critical evaluation to avoid maladaptation): • Provide financial assistance grants for home climate controls^a^ for those living in urban heat islands.	Local government, government department responsible for climate action
		Mapping of high-risk and densely populated areas for targeted prioritizing of heat-adaptation strategies.	Local government, government department responsible for climate action, national body responsible for environmental protection
		Erect emergency shelter venues during heatwaves.	Government department responsible for health
	Engineering	Ensure public spaces in towns and cities (e.g. libraries) have adequate climate controls^a^.	Local government
		Create public green and blue spaces in and around cities and towns and ensure adequate transportation infrastructure to enable access particularly from impoverished areas. A thorough critical evaluation of actions must be undertaken before implementation to avoid maladaptation.	Local government, government department responsible for transport and environmental protection, urban planners
Food Systems	Research	Further research is required in the following areas: • Direct and indirect impacts of heat on the food system in temperate climates and subsequent food and nutrition security • Evaluating adaptation mechanisms to protect livestock from excessive heat • Agricultural pest populations in a changing climate and an analysis and evaluation of pest control measures amongst farmers • Alternative heat-tolerant, nutritional food production with lower associated greenhouse gas emissions	Higher education institutions, research bodies, government department responsible for climate action, government department responsible for agriculture, funding bodies
	Engineering & policy	Heat-related mitigation and adaptation strategies should be utilized within the agricultural sector such as carbon sequestration and use of sustainable fertilizers.	Government department responsible for agriculture, local government
	Community	Sharing of knowledge between farmers on successful heat-related adaptation and mitigation strategies.	Government department responsible for agriculture, community
Health sector	Planning for preparedness	Access should be available for suitable parties to anonymised secondary data for analysis in identifying anticipated heat-related direct and indirect health outcomes, vulnerable groups, lag structures and health sector capacity and heat-specific resource needs.	Government department responsible for health, research institutions, funding bodies, statistics body
		Specific heat-related monitoring and surveillance should be implemented.	Government department responsible for health, research institutions, funding bodies, national body responsible for environmental protection
	Capacity building	Prepare / refresh frontline healthcare workers to manage the signs and symptoms of heat-related illnesses.	Government department responsible for health
	Engineering	Ensure back-up power supplies for critical infrastructure during hot days in healthcare services with low emission climate controls^a^.	Government department responsible for health
		Implement hospital site heat-smart landscaping to reduce the heat island effect.	Government department responsible for health, urban planners, national body responsible for environmental protection
		Energy audit should be conducted to analyse and reduce emissions from the health sector as well as to investigate possible over-heating of facilities during summer.	Government department responsible for health
	Research	Further research is needed in the following areas: • Analysis of secondary data for associations of disease burden in response to increased temperatures • Analysis of the burden of heat on health and subsequent health sector capacity during hot events	Government department responsible for health, research institutions, funding bodies, statistical bodies
General actions	Planning & policy	Develop and implement a heat-health alert service including pre-recorded heat-health messages.	Health promotion unit of the health sector
		Develop and implement early warning systems for heatwave predictions.	National meteorological body, national body responsible for environmental protection
		Develop a heat-health action plan in accordance with WHO guidelines. For successful implementation, the plan should include eight core elements: i) agreement on a lead body; ii) accurate and timely alert systems; iii) heat-related health information plan; iv) strategies to reduce indoor heat exposure; v) consideration of vulnerable groups; vi) health and social care system preparedness; vii) long-term urban planning and viii) real-time surveillance and evaluation. The heatwave plan for England would provide a good model that could be adapted locally and includes: i) strategic planning; ii) alert system; iii) preparedness; iv) communication; v) working with service providers; vi) engaging with the community; vii) monitoring and evaluation.	Government department responsible for health and climate action
		Develop emergency protocols for vulnerable sectors such as older people care homes, schools etc.	Local government, government department responsible for health
		Implement a “health and climate change in all policies” approach [108].	All government departments
		Provide a telephone hotline for specific heat-related emergencies including over the phone advice on preventing the escalation of heat strain to heat stroke.	Government department responsible for health
		Monitor water supply (including essential maintenance of public drinking fountains in preparation) and quality during summer and heatwaves.	National body responsible for environmental protection, government department responsible for climate action
	Engineering	Various engineering and planning controls could be developed as appropriate (after a critical evaluation to avoid maladaptation): • Invest in shade creation (site landscaping) and improve climate control^a^ in public buildings (e.g. libraries) and public transport vehicles with improved access for vulnerable groups to blue and green spaces. Opening hours to green and blue spaces could also be extended during hot days.	Local government, government department responsible for transport, national body responsible for the environment, urban planners
		• Collaborate with the housing sector to ensure appropriate housing design and retrofit with good ventilation in summer and insulation in winter.	Local government, urban planners, architects
	Communication & community	Develop and disseminate heat-related risk communication using traditional (e.g. pamphlets, advertisements, radio) and new media (e.g. social media, SMS, website) tools including what to do to avoid heat-related morbidity and mortality.	Health promotion unit of the health sector

Table Acronyms: *GPs* General practitioners, *NCDs* Non-communicable diseases, *WHO* World Health Organization

^a^Note: it is essential that the environmental impact of “climate controls” is minimal / negligible (e.g. zero emission)

## Conclusions

The aim of this manuscript was to review the evidence of heat-health vulnerability in a temperate region to determine the input from various responsible entities required. From the evidence presented by the review of literature, it is clear that there are many heat-vulnerable populations in Ireland yet there is a paucity of research. Additionally, there are many research and strategic gaps in an appropriate adaptation response to heat-health vulnerability in the region as seen by the recommendations provided in Table 1. The public and occupational health challenge of heat has not been adequately addressed in Ireland even though European vulnerability to heat, and subsequent cascading mental and physical health impacts, have been demonstrated in other temperate climates. Ignoring the issue of heat in public and occupational health policy places the Irish population at risk and is an environmental health injustice if the government and other responsible entities do not adequately respond, particularly given the high scientific consensus as to the certainty of future temperature rise for the region. It is imperative that more capacity and resources are put in place to avoid a threatening health crisis. We have provided a non-exhaustive list of recommendations and options for adaptation and mitigation planning across a range of vulnerable populations and sectors to allow for a specific and strategic intervention response. Furthermore, many of the proposed actions also have a dual benefit of increasing awareness of heat-health for the Irish population, even when travelling abroad to hotter parts of Europe or the world. In addition to the immediate recommendations provided, for future planning, given the multi-sectoral nature of the health impacts of heat and the extent of vulnerable populations and systems, we advocate that the Irish government should consider the strategy of a “health and climate change in all policies” approach as proposed by Godsmark et al. [108] and should develop and implement a public-health focussed heat-health action plan.

## Data Availability

The datasets analysed during the current study are available in the Google Trends repository (https://trends.google.com) and Met Eireann (met.ie/climate/available-data).

## Abbreviations

HSE: Health Service Executive
IPCC: Intergovernmental Panel on Climate Change
ISO: International Organization for Standardization
NCDs: Non-communicable diseases
NIOSH: National Institute for Occupational Health and Safety
RISFs: Regional internet search frequencies
UHI: Urban heat islands
WHO: World Health Organization
